# TIPE2 knockdown exacerbates isoflurane-induced postoperative cognitive impairment in mice by inducing activation of STAT3 and NF-κB signaling pathways

**DOI:** 10.1515/tnsci-2022-0282

**Published:** 2023-04-11

**Authors:** Rui Jian, Xin He

**Affiliations:** Department of Rehabilitation Medicine, The Affiliated Hospital of Southwest Medical University, Luzhou, Sichuan, 646000, China; Department of Anesthesiology, Xiangya Hospital, Central South University, Changsha, China; National Clinical Research Center for Geriatric Disorders, No. 87, Xiangya Road, Kaifu District, Changsha, Hunan, 410008, China

**Keywords:** POCD, TIPE2, STAT3, NF-κB, hippocampus, microglia

## Abstract

**Objective:**

Anesthetic exposure causes learning and memory impairment, the mechanisms of which remain unknown. It has been reported that tumor necrosis factor-α-inducer protein 8-like 2 (TIPE2) is a newly discovered immune negative regulator that is essential for maintaining immune homeostasis. This study aimed to examine the role of TIPE2 in isoflurane-induced postoperative cognitive decline (POCD).

**Methods:**

An AAV empty vector and AAV shTIPE2 vector for the knockdown of TIPE2 were injected into the dorsal hippocampus of mice. Mice were continuously exposed to 1.5% isoflurane followed by abdominal exploration. Behavioral tests including the open field test and fear conditioning test were performed on the third and fourth day post-operation. Apoptosis was detected by terminal deoxynucleotidyl-transferase-mediated dUTP nick end labeling staining. The kits were used to detect the activity of antioxidant enzymes. Inflammatory cytokine levels were detected by enzyme-linked immunosorbent assay. Signal transducer and activator of transcription 3 (STAT3) and nuclear factor-κB (NF-κB) signaling pathway activities were detected by western blotting.

**Results:**

TIPE2 expression increased after isoflurane anesthesia and surgery. TIPE2 deficiency aggravated cognitive impairment in mice and further caused apoptosis and oxidative stress in hippocampal neurons. TIPE2 deficiency induced microglial activation and increased secretion of proinflammatory cytokines. In addition, TIPE2 deficiency promoted STAT3 and NF-κB signaling activation induced by isoflurane anesthesia and after surgery.

**Conclusion:**

TIPE2 may play a neuroprotective role in POCD by regulating STAT3 and NF-κB pathways.

## 1 Introduction

Postoperative cognitive decline (POCD) is a recognized clinical phenomenon characterized by cognitive impairment in patients following anesthesia and surgery [1]. This complication is particularly common in the elderly [2]. POCD has attracted increased attention. In addition to its negative effects on cognitive domains such as memory and attention, POCD patients have increased mortality, decreased quality of life, and prolonged hospital stay [3]. However, the underlying mechanisms remain unclear and targeted interventions are not available yet.

It is well known that neuroinflammation and oxidative stress in the brain play a crucial role in the occurrence and progression of POCD [4]. Inflammation or oxidative stress from surgery can lead to neuroinflammation and damage in the brain, ultimately leading to cognitive impairment. Many pieces of evidence support that anesthetics used during surgery can also induce neurodegenerative symptoms [5,6,7]. Microglia are resident immune cells in the central nervous system and can be activated by anesthetics and surgical trauma that induces systemic inflammation [8,9].

Microglia are subsequently polarized to the M1 pro-inflammatory phenotype, inducing the secretion of pro-inflammatory cytokines such as IL-1, TNF-α, and IL-6 in the brain. The hippocampus, a center of memory and learning, widely expresses IL-6 and IL-8 receptors. Therefore, microglia switched to the M1 phenotype under pathological. In particular, it causes neurotoxic responses and synaptic dysfunction in the CA1 region of the hippocampus, ultimately leading to POCD [7].

Tumor necrosis factor-α-inducer protein 8-like 2 (TIPE2) is a newly discovered regulator of immune homeostasis in the TNFAIP8 family [10,11]. Current studies have shown that TIPE2 is associated with the progression of lung injury, hepatitis, asthma, and other diseases. Evidence suggests that overexpression of TIPE2 reduces the production of proinflammatory cytokine in the development of collagen-induced arthritis by inhibiting the activation of signal transducer and activator of transcription 3 (STAT3) and nuclear factor-κB (NF-κB) signaling pathways and alleviates arthritis in mice [12]. Overexpression of TIPE2 significantly prevented LPS-induced apoptosis of mice lung cells, blocked JNK phosphorylation, and nuclear translocation of NF-κB p65 [13]. In addition, TIPE2 plays an important role in alleviating nerve injury induced by middle cerebral artery occlusion. More inflammatory cells infiltrate into the ischemic hemisphere of TiPE2-deficient mice, and microglia are the main source of TIPE2 in the vulnerable region to ischemic injury [14]. The role of TIPE2 in postoperative cognitive dysfunction remains unclear. Therefore, we hypothesized that TIPE2 levels were upregulated after anesthesia and surgery. Abnormal expression of TIPE2 may be a self-protective mechanism to reduce inflammation and oxidative stress in hippocampal tissue by inhibiting microglia activation and activation of STAT3 and NF-κB signaling pathways, thereby improving POCD.

## 2 Materials and methods

### 2.1 Animals

Mice, 16-month-old male C57BL/6 (26–36 g), were purchased from the Model Animal Research Center of Nanjing University. Animals were kept under standard conditions. Constant temperature (19 to 22°C) and humidity (40 to 60%) and 12 h light/dark conditions were maintained in the feeding room. The mice were given free access to food and water, and acclimatization began a week earlier.


**Ethical approval:** The research related to animals use has been complied with all the relevant national regulations and institutional policies for the care and use of animals. All animal experiments in this study were performed in accordance with the Guide for the Care and approved by the animal ethics committee of the Affiliated Hospital of Southwest Medical University (Approval No. 202111115-003).

### 2.2 POCD model

The experiment was divided into four groups, with five mice in each group: (1) control, (2) surgery, (3) surgery + AAV, and (4) surgery + AAV shTIPE2. Abdominal exploration was performed under isoflurane anesthesia as described previously [3]. The mice were anesthetized for 30 min in a pre-filled chamber with 1.5% isoflurane oxygen, which was followed by surgery. A median abdominal incision of about 1 cm was made. The operator then probed the viscera, intestines, and muscle tissue. Sterile 4-0 chrome gut was used to close the inner peritoneum to the skin. The wound was bandaged with polysporin (Pfizer, USA) to prevent infection. The procedure, also under isoflurane anesthesia, lasted for 10 min. In the control group, neither anesthesia nor surgery was performed.

### 2.3 Virus and stereotaxic injection

The constructs were packaged into a chimeric AAV Serotype 8 vector. The AAV shTIPE2 vector knockdown of TIPE2 and the AAV TIPE2 vector for overexpressing TIPE2 were constructed by OBio Technology (Shanghai, China). AAV was delivered by bilateral stereotaxic injection into the CA1 region of the mouse brain following the mouse brain atlas (anteroposterior: 2.00 mm, lateral: ±1.50 mm, dorsoventral: −1.40 mm).

### 2.4 Behavioral tests

The open field test was performed on the third day after surgery. Each mouse was placed in the center of an open-air arena and was allowed to explore for 5 min, while a video tracking system automatically recorded the activity. The total distance traveled and the time spent in the center area were used to assess the mice’s motor activity.

The mice were subjected to the fear condition for 4 days after surgery. The mice were placed in the chamber for 2 min to acclimatize. This was followed by exposure to a sound stimulus (30 s, 70 dB, 3 kHz) and foot shock (2 s, 0.7 mA). This process was repeated twice with an interval of 60 s. The mice were then allowed in the room for another 30 s before returning to their home cage. The situational fear condition was assessed at 24 h post-training. Each mouse was returned to the same room, where they were allowed to explore for 5 min without tone or foot shock, and freezing time (no movement other than breathing) was recorded.

### 2.5 Western blot analysis

Mice were euthanized after completion of behavioral testing, and the hippocampal tissue was obtained by dissection. Hippocampal homogenates were lysed in cell buffer and subsequently subjected to western blot analysis as previously described [6]. The protein concentration was subsequently determined using the BCA Protein Assay Kit (Beyotime, China). Proteins were separated by SDS-PAGE (Beyotime, China) and transferred onto PVDF membranes (Millipore). The resulting membranes were blocked with 5% milk and incubated with specific primary antibodies overnight at 4°C. Primary antibodies included anti-TIPE2 (1:1,000), anti-β-actin (1:1,000), anti-CD11B (1:1,000), anti-p65 (1:1,000), anti-IκBα (1:1,000), anti-p-p65 (1:1,000), anti-p-IκBα (1:1,000), anti-STAT3 (1:1,000) and anti-p-STAT3 (1:2,000), and were purchased from CST. After rinsing, the membranes were incubated with a secondary antibody (Santa Cruz, USA) at room temperature for 2 h. The expression of related proteins was quantitatively analyzed using ImageJ.

### 2.6 Enzyme-linked immunosorbent assay (ELISA)

Hippocampal tissue homogenates were obtained, and the supernatant was collected by centrifugation at 10,000*g* for 10 min at 4°C. A Bradford protein assay of the supernatant was performed on each sample. TNF-α, IL-1β, and IL-6 levels were measured using the ELISA kit according to the manufacturer’s (R&D Systems) instructions and were normalized to the protein content.

### 2.7 Determination of the antioxidant enzyme activity and malondialdehyde content

SOD, CAT activity, and MDA content of the hippocampal tissue homogenate supernatant were determined using spectrophotometric kits according to the manufacturer’s instructions (Jiancheng Bioengineering Institute, China).

### 2.8 TUNEL assay

TUNEL assays were performed according to the protocol using an *in situ* cell death assay kit (Roche, Basel, Switzerland). Sections were re-stained with anti-NeuN antibody (1:200; Merck Millipore, Hong Kong, China) for 3 min. Tunel-positive cells were counted in six different microscopic fields from the CA1 region of each section of the hippocampus and the percentage was calculated.

### 2.9 Statistical analysis

All measurements for each experiment were made in triplicate. Data were expressed as mean ± SD. SPSS20.0 software was used for one-way analysis of variance, and Bonferroni post hoc test was used to compare differences between groups. Statistical significance was set at *p* < 0.05.

## 3 Results

### 3.1 Knockdown of TIPE2 exacerbates POCD

**Figure 1:**
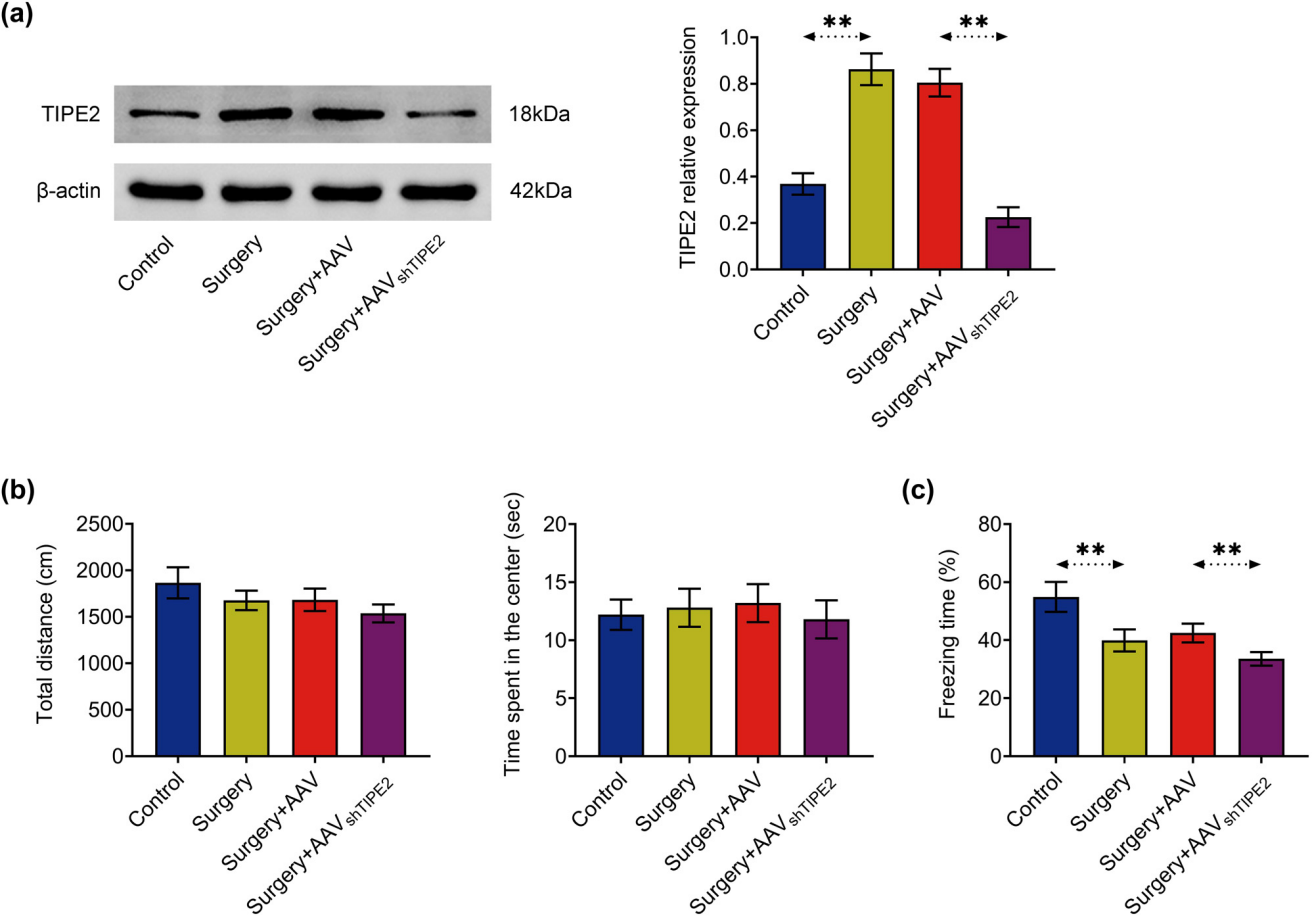
TIPE2 deficiency exacerbated isoflurane- and surgery-induced cognitive impairment in mice. (a) TIPE2 protein expression levels in the hippocampus of mice in each group were determined by western blotting. (b) On the third postoperative day, the travel distance and time spent in the central area when mice were allowed to freely explore the open-air arena for 5 min. (c) On the fourth postoperative day, the freezing time of fear conditioning in mice. **p < 0.01.

First, the expression levels of TIPE2 in different groups of mice were examined by western blotting (Figure 1a and Figure S1a). The results showed that TIPE2 levels were significantly increased in the hippocampus of mice after anesthesia and surgery compared to controls. AAV shTIPE2 transfection significantly inhibited the expression of TIPE2 in tissues. The mice were then subjected to behavioral tests. The total distance traveled by the mice and time spent in the center of the arena did not differ significantly between groups during the test (Figure 1b and Figure S1b). The results of the fear condition test showed that the freezing time of mice undergoing isoflurane and surgery was significantly shorter than that of controls, and the freezing time of mice with TIPE2 knocked down was further shortened after surgery (Figure 1c). Conversely, the freezing time was increased in mice with TIPE2 overexpression (Figure S1c).

### 3.2 Knockdown of TIPE2 enhances isoflurane- and surgery-induced apoptosis and oxidative stress in hippocampal neurons

**Figure 2:**
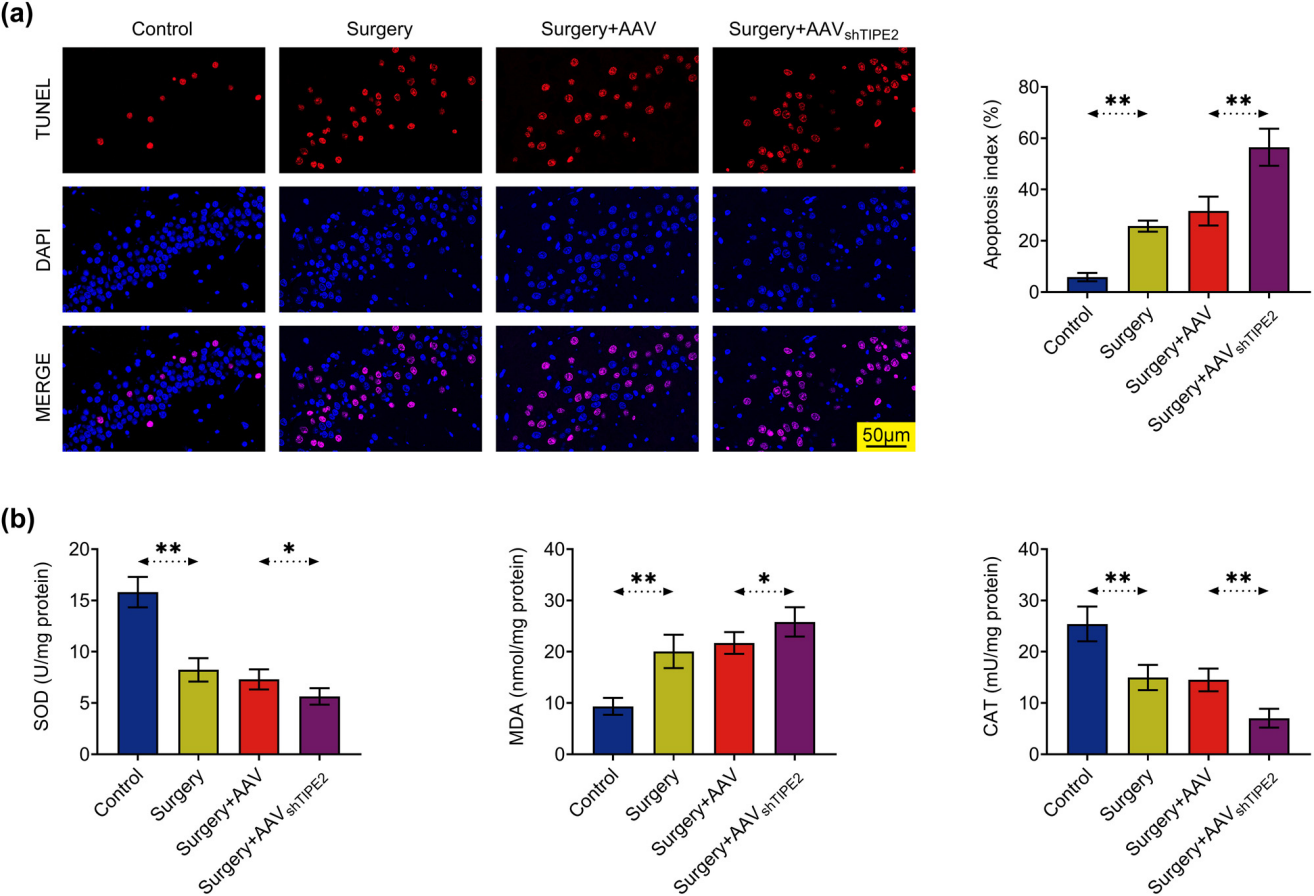
TIPE2 deficiency increased isoflurane- and surgery-induced apoptosis and oxidative stress in hippocampal neurons. (a) Tunel staining marked the apoptotic cells of hippocampal neurons, and the apoptosis index (%) was calculated. Scale bar  =  50 μm. (b) SOD, CAT activity, and MDA level in mice hippocampus. *p < 0.05; **p < 0.01.

As shown in Figure 2a, massive apoptosis of hippocampal neurons was induced after surgery. Compared with the surgery group, the number of apoptotic cells in the surgery + AAV shTIPE2 group was significantly increased. In addition, SOD, CAT activity, and MDA levels were also examined. The results showed that isoflurane and surgery induced a decrease in SOD and CAT activities and an increase in the MDA content in the hippocampus of mice compared to that in controls. Hippocampal oxidative stress was further aggravated in TIPE2-silenced mice (Figure 2b).

### 3.3 Knockdown of TIPE2 enhances isoflurane- and surgery-induced microglial activation and proinflammatory factor secretion

**Figure 3:**
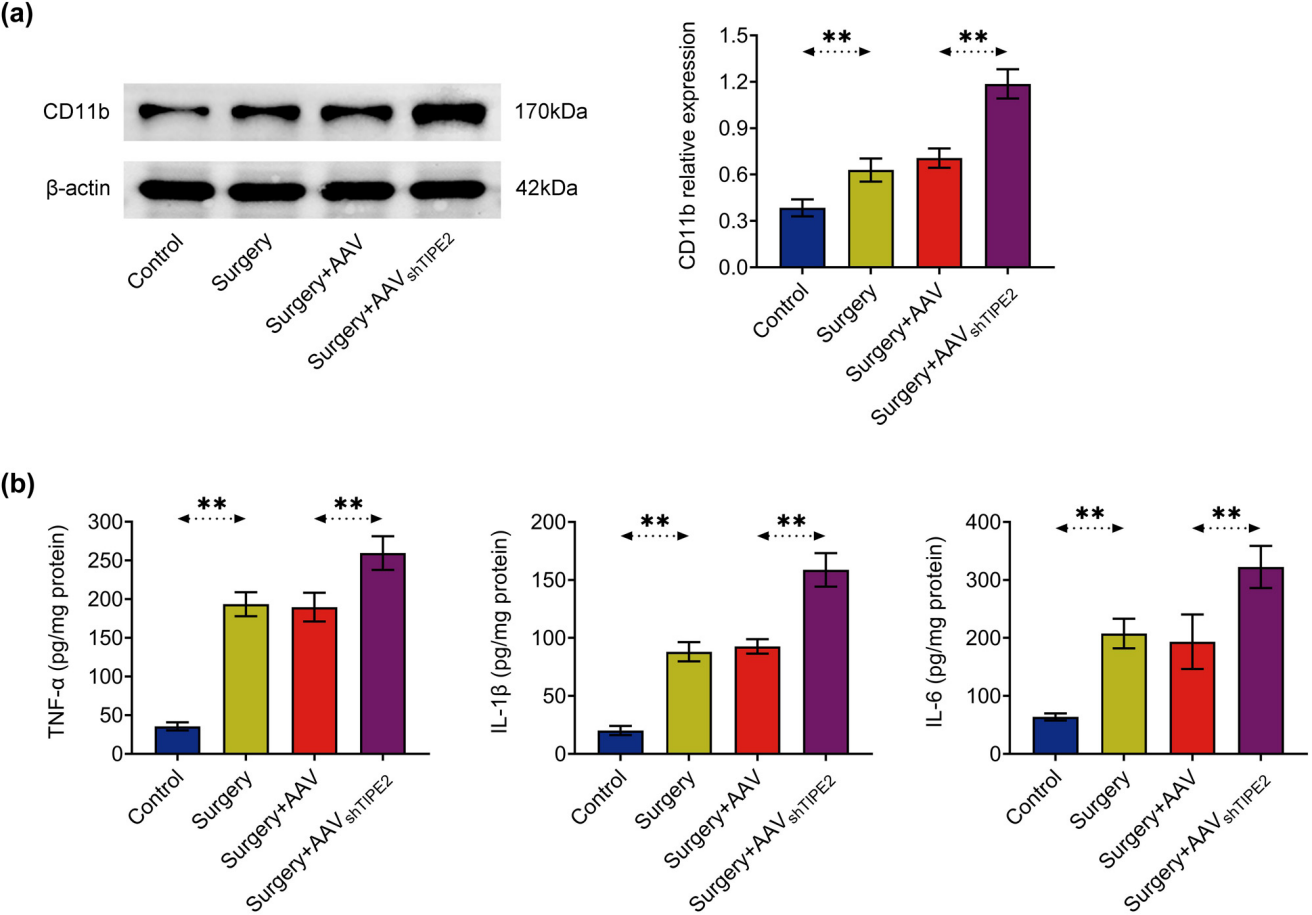
TIPE2 deficiency exacerbated isoflurane- and surgery-induced microglial activation and proinflammatory factor secretion. (a) Detection of the expression level of microglia activation marker CD11b in the hippocampus by western blotting. (b) Detection of inflammatory factors including TNF-α, IL-1β, and IL-6 levels in the hippocampus by ELISA. **p < 0.01.

Activated microglia are considered to be the main source of proinflammatory cytokines in the central nervous system [15]. Therefore, the expression level of CD11b was first detected by western blotting. Isoflurane and surgery significantly upregulated CD11b expression compared to the control (Figure 3a). The silencing of TIPE2 further exacerbated this phenomenon. As expected, ELISA results showed (Figure 3b) that the accumulation of proinflammatory cytokines including TNF-α, IL-1β, and IL-6 in the hippocampus of mice undergoing isoflurane exposure and surgery was significantly increased. The accumulation of TNF-α, IL-1β, and IL-6 in TIPE2-silenced mice was further increased.

### 3.4 Knockdown of TIPE2 activated the STAT3 and NF-κB signaling pathways

**Figure 4:**
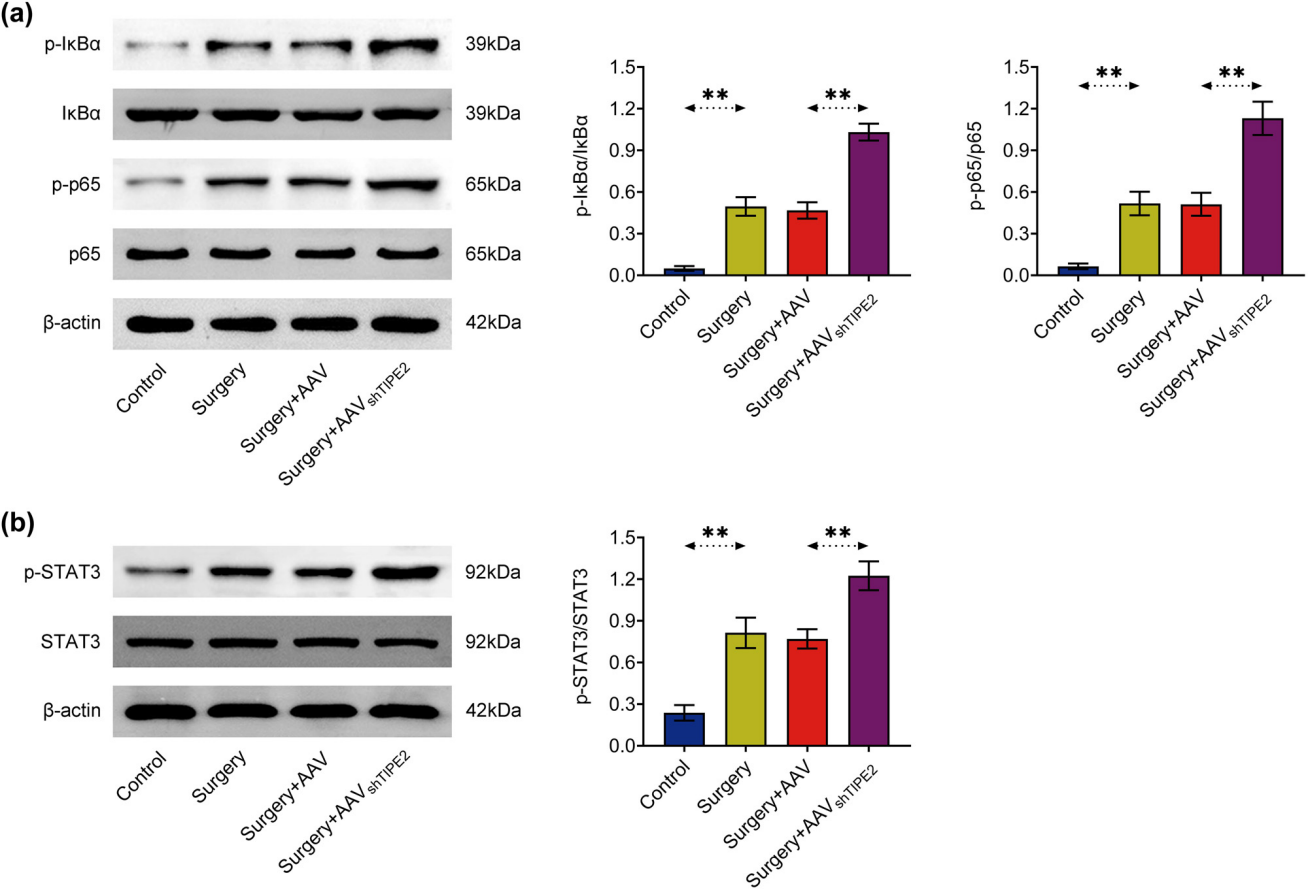
Knockdown of TIPE2 up-induced the activation of STAT3 and NF-κB signaling pathways. (a) Representative western blots of p65, IκBα, phosphorylated p65, and phosphorylated IκBα in the hippocampus. (b) Representative western blot of STAT3 and phosphorylated STAT3 in the hippocampus. **p < 0.01.

Next, we analyzed the effect of silencing TIPE2 on isoflurane- and surgery-induced STAT3 and NF-κB signaling pathways in mice hippocampus based on western blotting. Compared with the control, p-p65/p65 and p-IκBα/IκBα were significantly increased in the surgery group. Silencing TIPE2 induced further activation of the NF-κB signaling pathway (Figure 4a). Consistently, p-STAT3/STAT3 in the surgery group was also significantly higher than that in the control group, which was further increased in the TIPE2-silenced group (Figure 4b).

## 4 Discussion

Isoflurane is a widely used inhaled anesthetic in clinical practice. Clinical evidence suggests that prolonged isoflurane inhalation often causes long-term neurodevelopmental effects in infants and children as well as cognitive impairment in the elderly [16]. More than 25% of patients develop cognitive impairment after general anesthesia and surgery [17]. In elderly patients over 65 years old, the incidence of POCD has risen to nearly 40%, which may be related to age- and aging-related diseases (including neurodegenerative diseases, diabetes, and cardiovascular diseases) [18]. POCD can lead to life inconvenience, induce olfactory disorders, decrease short-term memory and concentration, and lead to worse prognosis and higher mortality [19,20]. A lot of research work has been done in recent decades but the mechanism of POCD remains to be determined.

In this study, we established an animal model of POCD by continuously exposing mice to 1.5% isoflurane and performing abdominal exploration. The results of the open field test and fear conditioning test showed that the motor activity of the model mice was not affected, but short-term memory decreased. Notably, western blot results showed that TIPE2 was differentially expressed between the control and model groups. TIPE2 has been reported to be aberrantly expressed in patients with various infectious and autoimmune diseases and is a key molecule in the prevention of inflammatory diseases [18]. TIPE2 deficiency causes more severe liver inflammation in mice [21]. TIPE2 overexpression can alleviate LPS-induced lung inflammation and lung cell apoptosis in mice [13]. Our study demonstrated that TIPE2 deficiency caused further aggravation of cognitive impairment in mice compared to control mice. This indicated that the abnormal expression of TIPE2 may be related to the progression of POCD but the mechanism of action is still unclear.

Hippocampus is a key brain region for spatial learning and memory, and disruption of the balance of antioxidant enzymes and reactive oxygen species, as well as neuroinflammation, causes damage to the hippocampus and further induces neuronal apoptosis, resulting in short-term and long-term cognitive impairment [22]. Previous *in vivo* experiments demonstrated the antioxidant and anti-apoptotic effects of TIPE2 in lung injury [13,23]. Our results demonstrated that the knockdown of TIPE2 exacerbates isoflurane- and surgery-induced apoptosis and oxidative stress in hippocampal neurons.

Previous studies have demonstrated the important role of TIPE2 in the immune system. TIPE2 can accelerate the differentiation of M2 macrophages by activating the PI3K signaling pathway, significantly reduce the expression of TNF-α, IL-6, and MCP-1, and play a protective role in atherosclerosis [24]. TIPE2 is involved in M1 macrophage and M1 cytokine-mediated neutrophilic airway inflammation in asthma through activation of the Nrf2/HO-1 signaling pathway [25]. Notably, in the brain tissue, microglia are the main source of TIPE2 [14]. Immune homeostasis is critical for the proper functioning of the immune system. Microglia are resident macrophages in the central nervous system [24]. High concentrations of proinflammatory mediators released by microglia with an M1-activated phenotype can cause neurotoxicity. Here, we investigated the role of TIPE2 in microglia activation and inflammatory cytokine secretion. We found that sustained isoflurane exposure induced upregulation of CD11b expression in the hippocampus of mice. CD11b expression was further upregulated in Tipe2-deficient mice. CD11b is a widely used classical marker protein and a marker of proinflammatory polarization in microglia cells. The results showed that surgery and anesthesia caused M1 polarization of microglia and TIPE2 deficiency caused increased activation of microglia. Activated microglia cells can upregulate the production of various pro-inflammatory factors. As expected, ELISA results showed that TNF-α, IL-1β, and IL-6 were accumulated in the hippocampus after surgery, and TIPE2 deficiency caused further exacerbation of neuroinflammation. It showed that abnormally expressed TIPE2 plays an important role in maintaining immune homeostasis after surgery.

Furthermore, to investigate the mechanism of action of TIPE2 in POCD, we performed western blotting. The results showed that the knockdown of TIPE2 further upregulated isoflurane- and surgery-induced activation of STAT3 and NF-κB signaling pathways. The STAT3 and NF-κB pathways are involved in multiple biological processes, including POCD. Phosphorylation and translocation of STAT3 in the hippocampus are enhanced after surgery, which activates microglia and astrocytes and induces neuroinflammation, DNA damage, and neuronal apoptosis, causing POCD [26]. NF-κB is a transcription factor that plays an important role in the inflammatory response of microglia and regulates multiple inflammatory cytokines, including IL-6, IL-1β, and TNF-α [27]. Inhibition of NF-κB can reduce neuroinflammation and secondary POCD [28].

Taken together, anesthesia and surgery induce upregulation of endogenous TIPE2 in the hippocampus. TIPE2 inhibits the activation of microglia by regulating the STAT3 and NF-κB pathways, improves the antioxidative and anti-inflammatory ability of hippocampal neurons, and inhibits apoptosis, thereby alleviating cognitive impairment. Our study demonstrates that TIPE2 played a neuroprotective role in POCD.

There are some limitations to our study. First, we examined levels of inflammation and oxidative stress in the hippocampal tissue of mice after anesthesia and surgery but did not study inflammation in other regions of the brain and throughout the body. Second, we observed relatively short-term behavioral and cognitive performance in mice through open field auditions and fear conditioning tests. In future studies, the Y maze and Morris water maze will be added to measure spatial cognition, and long-term relevant indicators will be detected in postoperative mice. In addition, whether TIPE2 directly regulates STAT3 and NF-κB pathways to relieve hippocampal inflammation and oxidative stress induced by anesthesia and surgery needs further investigation.

## Supplementary Material

Supplementary Figure
