# Supplementary material for: TIPE2 knockdown exacerbates isoflurane-induced postoperative cognitive impairment in mice by inducing activation of STAT3 and NF-κB signaling pathways
Source: Transl Neurosci. 2023 Apr 11;14(1):20220282. doi: 10.1515/tnsci-2022-0282 (PMC10105556; doi:10.1515/tnsci-2022-0282)
Supplement: Supplementary Figure [file tnsci-2022-0282-sm.pdf]

Supplementary material

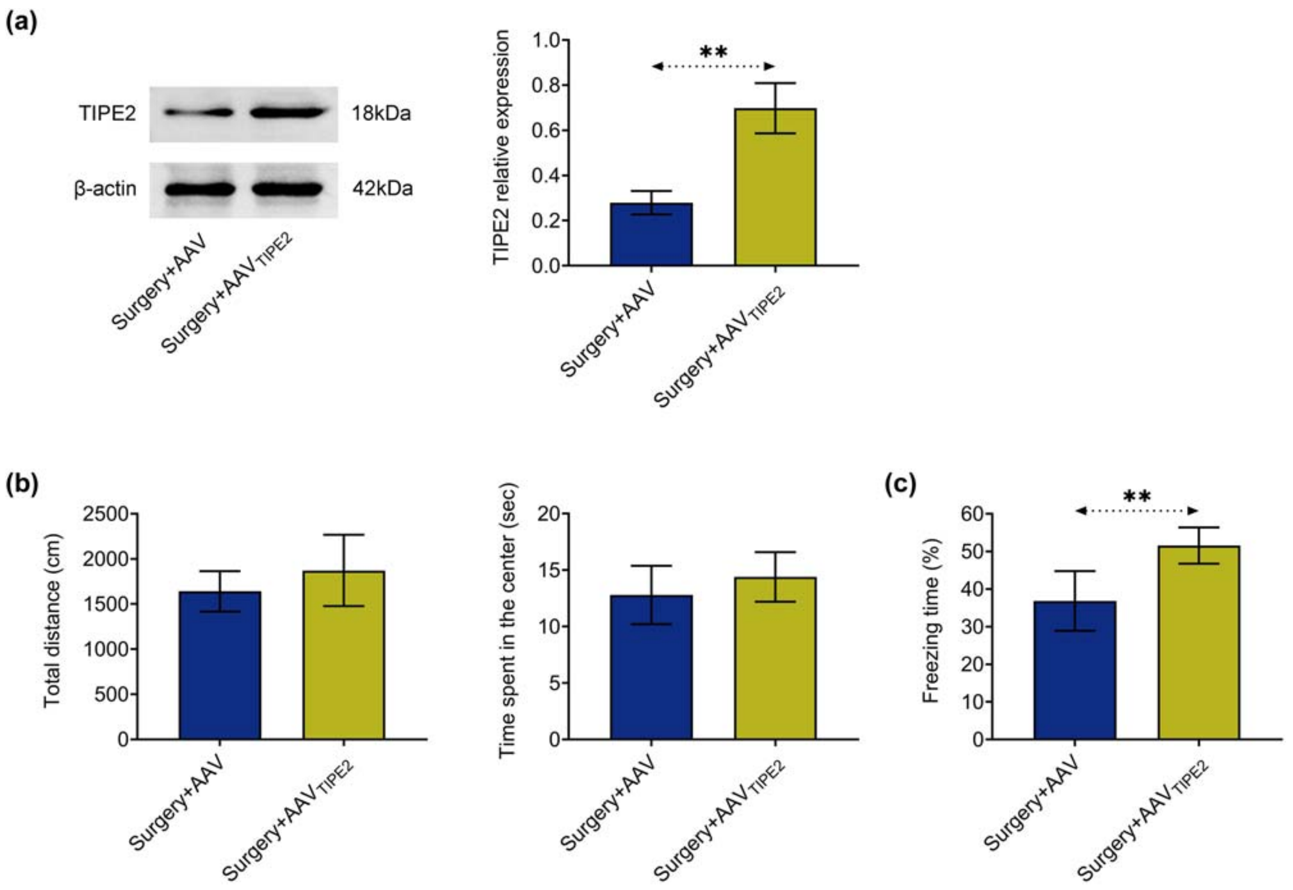

Figure S1: TIPE2 overexpression alleviated isoflurane- and surgery-induced cognitive impairment in mice. (a) TIPE2 protein expression levels were determined by western blotting. (b) On the third postoperative day, travel distance and time spent in the central area when mice were freely allowed to explore the open-air arena for 5 min. (c) On the fourth postoperative day, the freezing time of fear conditioning in mice. **$p < 0.01$.
